# Nucleic Acid Nanoparticles at a Crossroads of Vaccines and Immunotherapies

**DOI:** 10.3390/molecules24244620

**Published:** 2019-12-17

**Authors:** Marina A. Dobrovolskaia

**Affiliations:** Nanotechnology Characterization Lab, Frederick National Laboratory for Cancer Research sponsored by the National Cancer Institute, Frederick, MD 21702, USA; marina@mail.nih.gov; Tel.: +01-301-228-4935

**Keywords:** nanoparticles, safety, immunotoxicity, in vitro, in vivo, antigen-presenting cells, vaccines, adjuvants, antibody, immunotherapy, nanotechnology, preclinical, translation

## Abstract

Vaccines and immunotherapies involve a variety of technologies and act through different mechanisms to achieve a common goal, which is to optimize the immune response against an antigen. The antigen could be a molecule expressed on a pathogen (e.g., a disease-causing bacterium, a virus or another microorganism), abnormal or damaged host cells (e.g., cancer cells), environmental agent (e.g., nicotine from a tobacco smoke), or an allergen (e.g., pollen or food protein). Immunogenic vaccines and therapies optimize the immune response to improve the eradication of the pathogen or damaged cells. In contrast, tolerogenic vaccines and therapies retrain or blunt the immune response to antigens, which are recognized by the immune system as harmful to the host. To optimize the immune response to either improve the immunogenicity or induce tolerance, researchers employ different routes of administration, antigen-delivery systems, and adjuvants. Nanocarriers and adjuvants are of particular interest to the fields of vaccines and immunotherapy as they allow for targeted delivery of the antigens and direct the immune response against these antigens in desirable direction (i.e., to either enhance immunogenicity or induce tolerance). Recently, nanoparticles gained particular attention as antigen carriers and adjuvants. This review focuses on a particular subclass of nanoparticles, which are made of nucleic acids, so-called nucleic acid nanoparticles or NANPs. Immunological properties of these novel materials and considerations for their clinical translation are discussed.

## 1. Introduction

The major physiological function of the immune system is the protection of the host from pathogens. In some infections, the immune response to a pathogen is so robust that in the process of pathogen elimination, it also causes damage to healthy tissues [1,2]. The purpose of vaccines, therefore, is to stimulate immunity to pathogens without causing damage to the host. The immune system’s function, however, extends beyond fighting pathogens and is involved in many processes and normal function of non-immune tissues, and organs [3]. Healthy immune system operation is traditionally viewed as a constant and well-controlled balance between activation and suppression [4]. When a tight control over the balance is lost, and the function shifts toward the activation, the consequences to the host include an inflammatory disease or autoimmunity. Likewise, when the balance shifts towards suppression, it weakens the host’s resistance to malignant transformed cells and microbes resulting in cancer and recurrent infections [4]. As such, traditional treatment of inflammatory and autoimmune diseases involve anti-inflammatory and immunosuppressive drugs; while immune-stimulatory treatments intend to improve the resistance to tumors and infections [5,6]. Newer data suggest that the imbalance between positive and negative regulations of the immune effector and regulatory functions contribute to immune dysfunction disorders such as autoimmunity, and warrant consideration of novel treatments that focus on controlling the activity of regulatory and effector cells [7]. The goal of the immunotherapy, therefore, is to restore and maintain the immune homeostasis to provide adequate response of the host to the dynamic internal and external environment. 

**Figure 1:**
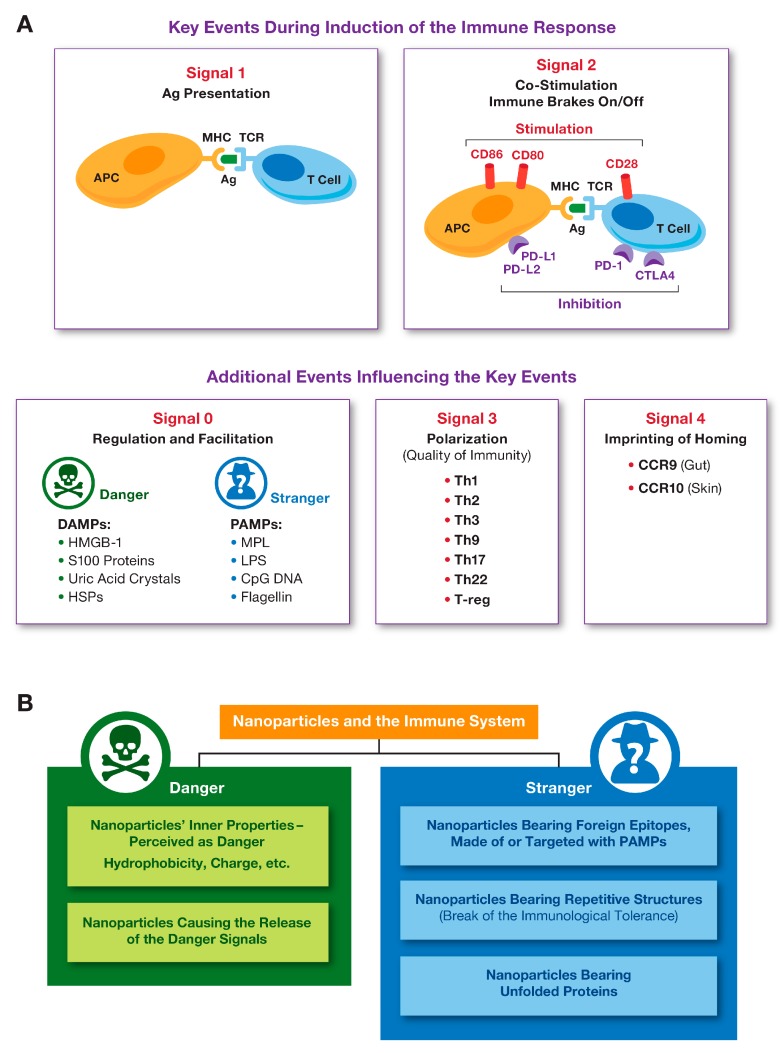
Key events during the induction of the immune response, and the role of nanoparticles in the immunity. (**A**) The two key events and other prerequisites of the optimal immune response are highlighted. The stimulation signal is provided through the interaction between co-stimulatory molecules (CD80, CD86) on the APC surface and CD28 on the T-cell surface; the inhibition occurs when PD-1 on the T-cell interacts with PD-L1 on the APC, or when CTLA4 on the T-cell interacts with CD80 and CD86 on the APC. (**B**) Nanoparticles can be perceived as either stranger or danger by the immune cells, depending on their physicochemical properties, cargo, and external environment (e.g., protein corona), which is also determined by the particle’s PCP.

The immune response against an antigen involves various cell types that interact via multiple pathways culminating with the formation of the antigen-specific T-cell clone, an antibody-producing plasma B-cell clone as well as T- and B-cell memory. The two key events required for this response to occur are known as signal 1, which is antigen presentation by an antigen-presenting cell (APC) to a T-cell via MHC I or MCH II pathway, and signal 2, which is the interaction between the APC’s co-stimulatory molecules (CD80 and D86) and the T-cell positive stimulation receptor CD28. A T-cell, which received both signal 1 and signal 2 from APC, becomes activated and proliferates to produce the antigen (Ag) specific T-cell clone (Figure 1A). Antigen-presentation via MHCI and MHCII pathways results in the activation of CD8+ (cytotoxic) and CD4+ (helper) antigen-specific T-cells, respectively. To provide the negative regulation and prevent overt T-cell activation, PD-1 on the surface of the activated T-cells and PD-L1 on the surface of APC, as well as CTLA4 on the surface of the activated T-cells and co-stimulatory molecules (CD80 and D86) on the APC interact to stimulate inhibitory pathways that act to quench the activation (Figure 1A). This model of the induction of the specific and controlled immune response is also known as two-signal hypothesis [8]. However, PD-1, PD-L1, and CTLA4 are not the only negative regulators of T-cell activation. Other inhibitory receptors (LAG-3, TIM-3, TIGIT) and ligands (B7-H3, B7-H4, and B7-H5) have been recently described; the understanding of their mechanism of action and opportunities for pharmacological intervention are currently underway [9]. It is also well established that a variety of responses of the antigen-presenting cells and other components of the innate immune system (e.g., complement system) to the presence of foreign antigens exist and contribute to the development of the specific adaptive immunity. For example, the activation and conditioning of APC to better perform their antigen-presenting function depends on so-called Pathogen Associated Molecular Patterns (PAMPs) expressed by the microbes and Danger Associated Molecular Patterns (DAMPs) produced as a result of host damage by either microbes or internal and external stimuli [10]. These interactions between PAMPs, DAMPs, and APCs are often referred to as Signal 0 (Figure 1A). Activated by PAMPs and DAMPs, APCs express higher levels of MHC II and co-stimulatory molecules as well as secrete cytokines which promote the production of antibodies by B-cells (e.g., IL-6), differentiation of naive CD4+ T-cells to Th1 (e.g., IL-12) or Th17 (e.g., IL-1, IL6 and IL-23), and survival of CD8+ memory T-cells (e.g., IL-15) [8]. Activation of plasma complement proteins contributes to the activation of APCs, improves B-cell and T-cell activation [8], while intra-cellular complement activation controls CD4+ T-cell differentiation to Th1 lymphocytes and contributes to the maintenance of tolerance to self-antigens [11]. The quality of immunity depends on the type of T-helper and suppressor (T-regs) cells. The messenger molecules including but not limited to cytokines, chemokines, and growth factors influence the quality of the immune response and are aften cited as Signal 3 (Figure 1A). The imprinting of the immune cell homing is regulated by the chemokines and their receptors such as CCR9, expressed in the gut, and CCR10, expressed in the skin [8]. These signals are often called Signal 4 (Figure 1A).

Adjuvants are substances that improve the immune response. Many known adjuvants (e.g., alum, Complete Freund Adjuvant or CFA, CpG DNA, and monophosphoryl lipid A) act as strangers or cause damage to induce danger signals. Adjuvants used in vaccines and immunotherapies have diverse molecular structures and are required to stimulate the immune cells at levels that are just enough to achieve the desired type of immune response but not too strong to avoid adverse effects [12]. It is very challenging to select and develop adjuvants because the magnitude of the immune cells’ response to molecules with the same molecular structure may vary dramatically between different individuals [13]. The polymorphism of genes encoding immune receptors, transcription factors and signaling molecules involved in the immune responses is responsible for the diversity of individual responses to adjuvants among humans. 

Nanoparticles can be perceived as either stranger or danger by the immune cells. As such, they serve as important components of Signal 0 (Figure 1B). The immune recognition of nanoparticles depends on their physicochemical properties (PCP), cargo, and external environment (e.g., protein corona), which is also influenced by the nanoparticle’s PCP [14,15]. The relationship between nanoparticle structures and their effects on the immune system has been extensively discussed elsewhere [14,15,16,17].

Most of the currently available vaccines induce humoral immunity, i.e., the production of the antibody specific to the antigen. There is an increased interest in so-called therapeutic vaccines, which aim at inducing the antigen-specific cytotoxic T-lymphocytes. These vaccines are of particular importance to cancer therapy [18,19,20]. 

The efficacy of microbial vaccines depends on several properties of disease-causing microbes, which include the microbe’s ability to establish latency, undergo antigenic genetic variability and impact the host’s immune system. Some microbes, e.g., human immunodeficiency virus, are notoriously difficult to vaccinate against because they tend to become latent, endure constant change in the surface antigen repertoire and inhibit the function of T-helper lymphocytes, which are crucial for the effective immune response [21]. Different types of vaccines are being developed and include the following categories: attenuated and inactivated microbes, recombinant microbes, purified antigen or so-called subunit vaccines, synthetic antigen vaccines, RNA and DNA vaccines [8,22,23]. All of these vaccines typically require an adjuvant. The new generation vaccines, which include subunit, synthetic antigens, RNA and DNA vaccines often also require a delivery carrier. Nanotechnology carriers are popular in the vaccine field due to their ability to both deliver the antigens to the antigen-presenting cells and act as adjuvants that activate APCs to express co-stimulatory molecules, upregulate their MHCII expression and produce cytokines educating optimal T-cell responses [24,25,26,27]. 

While immunotherapy in general, refers to the therapy that restores normal function of the immune system by providing either activation (e.g., in a disease caused by abnormally inhibited immune system) or inhibition (e.g., in a disease caused by abnormal activation of the immune system), this term is more used and gained its popularity in the field of cancer therapy, where the immunotherapy is used to restore the activity of anergic T-cells and exhausted APCs [28,29,30,31,32,33]. The cancer immunotherapeutics include check-point inhibitor blockade agents (e.g., anti-CTLA4, anti-PD-1, and anti-PD-L1), M2/M1 macrophage repolarizing agents (e.g., resiquimod), cancer antigen-specific chimeric antigen receptor (CAR) T-cells, and adoptive transfer of tumor-infiltrating T-lymphocytes (TILs) to name the few [28,29,30,31,32,33].

The benefits of nanotechnology for the field of vaccine and immunotherapies have been extensively discussed in the literature [28,32]. The recent advances in vaccines and immunotherapies have also received broad attention in several recent publications [29,30]. Herein, I will focus on the immunological properties of therapeutic nucleic acids and, particularly, nucleic acid nanoparticles (NANPs) in the context of vaccines and immunotherapies.

## 2. Therapeutic Nucleic Acids

Therapeutic Nucleic acids (TNAs) is a large family of compounds that includes diverse nucleic-acid based materials with different molecular weight (e.g., small and macromolecular TNAs), composition (e.g., DNA, RNA, and their chemical analogs), and geometry (e.g., linear, planar and 3-D). Some of these materials have been used in medicine for a long time. For example, small molecule nucleotide and nucleoside analogs (NNA) are used for the therapy of viral infections and cancer due to their ability to interfere with DNA replication and repair, transcription, and stability of DNA or RNA either directly or by affecting the function of enzymes, receptors and structural proteins involved in these processes [34]. 

**Figure 2:**
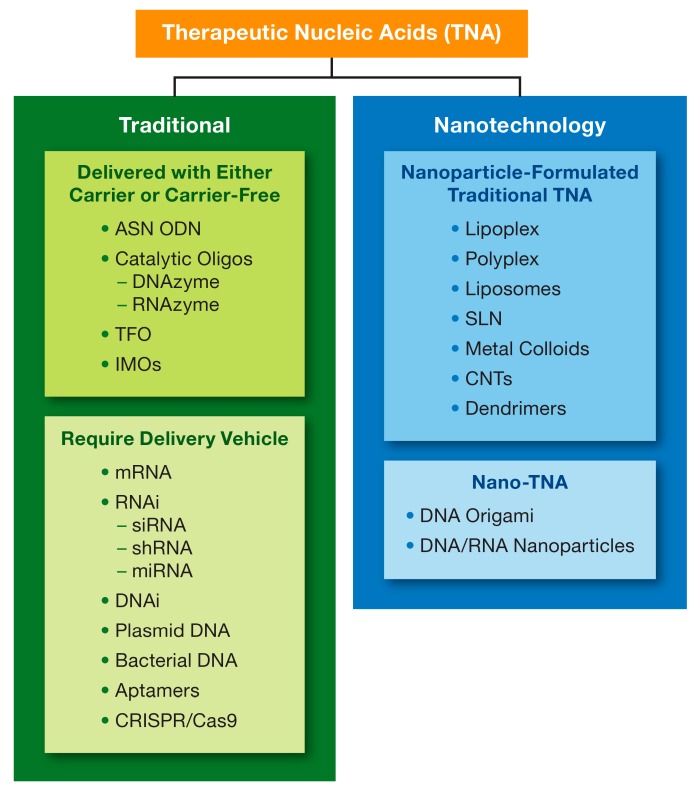
Type of therapeutic nucleic acids. A wide variety of therapeutic nucleic acids (TNAs) is described in the literature and include both short oligonucleotides made of DNA or RNA (e.g., ASN ODN), and macromolecular complex structures (e.g., CRISPR gRNA and Cas9 mRNA). Nanotechnology is either used for the delivery of some TNAs (nanoparticle-formulated TNAs) using carriers listed in the top blue box or to program and fold nucleic acids into various geometric shapes (Nano-TNAs). ASN = anti-sense; ODN = oligodeoxyribonucleotide; DNA = deoxyribonucleic acid; RNA = ribonucleic acid; siRNA = small interfering RNA; RNAi = RNA interference; shRNA = short-hairpin RNA; miRNA = microRNA; TFO = triplex-forming oligonucleotides; IMOs = immunomodulatory oligonucleotide; CNT = carbon nanotubes; CRISPR = clustered regularly interspaced short palindromic repeats; SLN = solid lipid nanoparticles.

The earlier versions of macromolecular TNAs, including aptamers, antisense oligonucleotides (ASN), triplex-forming oligodeoxyribonucleotides, catalytic oligonucleotides, inhibitory RNA (RNAi) and DNA (DNAi), CpG oligonucleotides, and regulatory RNAs (miRNA) have also been used in biomedical applications due to their ability to affect the expression of genes at DNA, RNA and protein levels [35,36,37]. For the purposes of this review, I will refer to them as traditional TNAs, wherein “traditional” implies a greater experience of research community and better knowledge about these materials in comparison to other more recent constructs and relatively simple design principles. The newer generations of the TNAs include larger nucleic acid constructs such as mRNA, CRISPR sgRNA, which require a carrier for intracellular delivery, and rationally designed multistranded assemblies of nucleic acids such as DNA origami and Nucleic Acid Nanoparticles (NANPs), which may or may not require a carrier depending on their applications. For the purposes of this review, I will refer to these materials as Nanotechnology TNA, where “nanotechnology” implies either delivery of traditional or new TNAs using a nanoparticle carrier (nanotechnology-formulated TNA) or a nanosized structure formed through the self-assembly of engineered oligonucleotides into complex 3D objects with defined topologies and PCP (nano-TNA) (Figure 2). Both traditional and newer, nanotechnology-based TNAs, are equally complex and sophisticated, have unique properties, and require thorough analysis in the context of their intended biomedical use. 

Many challenges regarding the pharmacokinetics, toxicology, and stability in biological matrices of the traditional TNAs have been resolved by chemical modifications of nucleotides’ backbone and sequences, as well as optimization of dosing regimen and the route of administration of traditional TNAs [38,39,40,41,42]. Additional strategies have also been proposed [43]. These materials were tested as is and after complexation with a delivery carrier. Commonly considered delivery carriers include liposomes, lipoplexes, polyplexes, etc. (Figure 2). Many of these concepts entered clinical trials, and some received regulatory approval. The approved for clinical use TNAs include fomivirsen (Vitravene^®^), an ASN intended for therapy of HIV-related cytomegalovirus infection; mipomersen (KYNAMRO^®^), another ASN for the treatment of homozygous familial hypercholesterolemia; pegaptanib (Macugen^®^), a DNA aptamer for the treatment of age-related macular degeneration; patisiran (Onpattro^®^) and inotersen (TEGSEDI^®^), an siRNA and ASN, respectively, for the treatment of polyneuropathy developed as a consequence of hereditary disease, transthyretin-mediated amyloidosis; givasiran (GIVLAARI^®^), a GalNAc formulated siRNA for the treatment of acute hepatic porphyria. These formulations are administered to patients through either the non-systemic routes, i.e., intravitreous and subcutaneous, or slow infusion to avoid the immediate interaction with and recognition by the innate immune cells and complement proteins in the blood, which activation by these materials may lead to severe immune-mediated adverse effects in sensitive individuals. Injection site reactions may occur in some patients. In the case of s.c. administered givasiran, for example, the frequency of the injection site reactions was as high as 25% [44]. One of the approved materials, i.e., patisiran (Onpattro^®^), employes a nanoparticle carrier.

While nanoparticle-formulated mRNA and CRISPR sgRNA approached the stage of clinical testing, none of these materials are approved for clinical use. The nano-TNA is a relatively new technology. These sophisticated materials are still in the stage of discovery and preclinical development. Many properties required to classify these materials as drug products are still unknown and may vary tremendously from one nano-TNA to another. Yet, currently available data suggest that nano-TNA may form a separate class of drug products distinct from traditional TNAs, small molecules, and biologics [45].

## 3. Nucleic Acid Nanoparticles (NANPs)

### 3.1. General Overview

Individual DNAs, RNAs or their chemical analogs can be computationally designed to assemble in a controlled fashion into NANPs with various composition, geometry, and functionality. Available strategies for forming these complex nanostructures among others include the extensive use of available RNA tertiary motifs, an approach called RNA architectonics, where all individual RNA motifs are considered to be “Lego blocks” and can be recombined in a particular way to address the required geometry of NANP [46,47,48,49,50,51,52,53,54,55,56,57,58]. Another powerful approach, called DNA origami, allows for controlled formation of DNA nanostructures by using longer ssDNAs stapled by shorter oligos [59,60,61,62,63,64]. Besides other numerous benefits, such as precise control over their size, charge and composition, NANPs have been shown to be stable under external conditions (e.g., radiation, temperature, nucleases), which typically would degrade traditional oligonucleotides [65,66,67,68,69]. More importantly, NANPs technology is flexible and allows adding functionalities and swap individual nucleotides without altering the entire nanoparticle assembly [70]. As such, NANPs can be designed to form non-functional scaffolds, which are typically made of DNA, RNA or DNA-RNA hybrid oligonucleotides not-specific to any particular target gene, use these scaffolds to simultaneously deliver multiple different gene-specific therapeutic oligonucleotides (e.g., siRNAs or miRNAs) and create more sophisticated materials with conditionally activatable split functionalities [46,48,53,54,65,66,67,68,70,71,72,73,74,75,76,77,78,79,80,81,82,83,84,85]. A co-delivery of individual non-functional scaffolds is required for applications involving NANPs with split functionalities and have been demonstrated in biological systems both in vitro and in vivo [53,73,74,77,82]. 

There is also an increasing trend in considering the NANP scaffolds for the controlled immunomodulation, wherein control is provided by several means including but not limited to modifications in the nanoparticle physicochemical properties (e.g., composition, size, shape, sequence complementarity, connectivity), delivery vehicle, dose and route of administration [76,81,82,86,87]. 

### 3.2. Delivery and Distribution to and within Tissues and Cells

**Figure 3:**
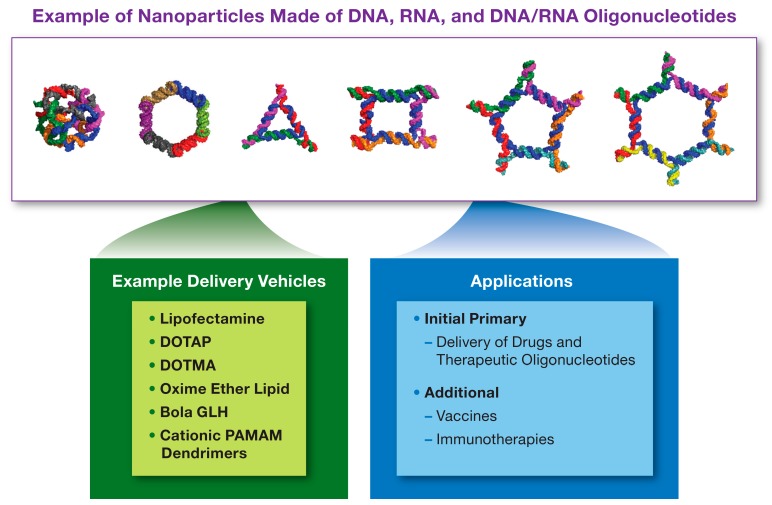
Nucleic Acid Nanoparticles. Nucleic Acid Nanoparticles (NANPs) are made of DNA, RNA or hybrid DNA/RNA oligonucleotides, which assemble into nanostructures with different sizes, geometric shapes, sequence complementarities, and other functional modalities. Some examples of NANPs are shown above. The examples of platforms used to deliver NANPs to cells and tissues are listed in the green box, while their applications in pharmaceutical field are summarized in the blue box. DOTAP = 1,2-Dioleoyl-3-trimethylammonium propane; DOTMA = *N*-[1-(2,3-dioleyloxy)propyl]-*N*,*N*,N-trimethylammonium chloride; PAMAM = polyamidoamine; GLH = bolaamphiphiles.

NANPs’ delivery routes described in the literature include both local and systemic administration. For local administration, commonly used delivery methods include the injection of NANPs directly into the tissue of interest (e.g., subconjunctival and intratumoral). When a systemic delivery is preferred, NANPs are injected intravenousely [66,67,73,78,88]. NANPs stability in biological matrices can be tuned by optimizing their structural complexity, and chemically modifying the backbone and individual nucleotides similar to the modifications described for traditional nucleic acids [89]. Further optimization of stability, as well as targeted delivery to the cells or tissues of interest, can be achieved by complexing these materials with delivery carriers [90]. Cationic lipids, bolaamphiphiles, poly- and lipoplexes, and inorganic nanoparticles have been described in the literature, as most common carriers used for NANPs [72,73,80,91,92,93,94], as seen in Figure 3.

To study tissue distribution, NANPs are commonly labeled with fluorescent probes. For example, Guo’s lab reported that unlike many other nanoparticle platforms, systemically administered three-way junction (3WJ) pRNA nanoparticles do not accumulate in healthy tissues (liver, spleen, lungs, and kidney) and distribute to the target tissue within the first hour after an injection [66,95,96,97]. It was hypothesized that due to the sizes under 10 nm pRNA nanoparticles would clear via renal route [97]. Tetrahedron-forming NANPs used for the delivery of siRNA were demonstrated in another study to have a short (~24.2 min) plasma half-life; these particles distributed to the tumor and were observed in the kidney suggesting renal clearance [98]. These findings are in agreement with clinical data reported with traditional TNA [99]. Afonin’s lab reported that hybrid NANPs accumulated in tumors but were also detected in the heart, lungs, liver, spleen, kidney, brain, and bladder [73]. The findings from this group also support renal clearance as the main route of NANPs elimination from the body. The accumulation of NANPs in other tissues observed in this study may be explained by the higher dose and greater stability of these NANPs, as opposed to the materials investigated in other studies.

Locally delivered NANPs distribute through the lymphatic system. Guo’s lab compared the distribution of 3WJ and 4 WJ NANPs to sclera and retina after topical and subconjunctival administration and found that only 4WJ NANPs reach retinal cells while other studied NANPs distribute to cornea, sclera, and conjunctiva [78]. 

Targeted delivery of NANPs to cells of interest could be achieved by adding aptamers to their structures or by providing delivery vehicles. For example, Shu et al. demonstrated the delivery of 3WJ nanoparticles into cancer cells overexpressing folate receptor by attaching the folate to these NANPs [66]. In another study, an aptamer binding to the HIV gp120 protein was used to target pRNA nanoparticles to the HIVgp120-expressing cells [100]. A recent study in healthy human peripheral blood mononuclear cells (PBMCs) demonstrated that NANPs of various compositions and geometric shapes are taken up by monocytes via endolysosomal pathway after the complexation with lipofectamine, but remain in the extracellular space without such complexation [87]. Interestingly, these data contrast NANPs to traditional TNAs that do not require a carrier and are internalized via pino- and podocytosis pathways [40]. Respectively, the ODN2216, an oligonucleotide used in this study as the positive control, did not require the carrier to enter the cells and elicit an inflammatory response [87]. Another interesting finding from this study is that the uptake of NANPs-lipofectamine complex is mediated by the scavenger receptor [87]. Scavenger receptors on mononuclear phagocytic cells are known for their role in clearing up polyanionic materials, including self-DNA released from dead cells and therapeutic oligonucleotides [101]. It is very interesting that NANPs are not captured by these receptors until they are concentrated and presented by the lipid carrier. The secondary structure of these materials may be responsible for this phenomenon. These findings also suggest that one can control immunological recognition of NANPs by directing them to or bypassing the endolysosomal pathway via complexation with different carriers. More research is obviously needed in this area. Hybrid NANPs were also shown to accumulate in the endosomal compartment of cancer cells [73]. Similar to the PBMC study by Hong et al., [87] NANPs, in this case, were also complexed with lipofectamine carrier prior to the addition to the cancer cells; the role of the receptor(s) involved in the uptake, however, was not investigated [73].

### 3.3. Toxicity 

Despite the documented fact that NANPs can distribute to the off-target organs (brain, kidney, liver, and spleen), their toxicity to these organs is not fully understood [73]. The available data suggest that NANPs are well tolerated by animals at the doses required to achieve desired therapeutic effects [66,96,97]. However, dose range-finding studies to establish the maximum tolerated dose (MTD) for individual NANPs have not been conducted. It would be interesting to see how MTD of the same NANP depends on the route of administration, a carrier (if the carrier is used) and dosing regimen. So far, all in vivo studies investigating NANPs general toxicity were conducted in mice. It would be interesting to see how these materials are tolerated by other rodents (e.g., rats) and different animal species (e.g., dogs and non-human primates). Potential developmental and reproductive toxicity as well as genotoxicity of NANPs is a gray area and needs thorough investigation. 

### 3.4. Immunological Properties

Although some attempts were made to tap into the immunological properties of individual NANPs [49,66,73,96,97,102,103], the systematic structure-activity relationship using primary human immune cells and a library of NANPs with various structures, composition, method of production and design, were unknown until recently [87]. The earlier studies identified several patterns of interest to the immunological applications of NANPs. They demonstrated that a short exposure to 3WJ pRNA nanoparticles does not induce inflammation in cultured cell lines [97]; the same 3WJ pRNA do not activate expression of Toll-Like Receptors (TLR3, TLR7, and TLR9) in human monocytes [97]; DNA/RNA hybrid cubes induce some cytokines and type I interferons in human PBMCs depending on the amount of RNA present in their structures [72]; planar RNA NANPs induce pro-inflammatory cytokines in murine RAW264.7 cells in the shape-dependent manner [103]; when NANPs were designed to deliver CpG DNA oligonucleotides or antisense oligonucleotides, their proinflammatory properties appear to increase [82,103]. However, the main shortcomings of these studies that made it difficult to compare between the results generated by the individual research groups were the use of different models, different endpoints, different biomarkers of the immune response, and NANPs designed by different algorithms and produced by different techniques. In order to advance the field of therapeutic nucleic acid nanotechnology, a recent pioneering study investigated a library of 24 NANPs that had different sizes, 3D conformation (planar, globular, and fibrous), connectivity, sequence complementarity, and made of either RNA or DNA [87]. To make the study applicable to and representative of the entire field of nucleic acid nanotechnology, the authors constructed a library using the design and technology developed and published by different research groups. This approach allowed the comparison of different NANPs side-by-side under equivalent experimental conditions and in the same model, using the same end-points and biomarkers of immunostimulation. This comprehensive study identified that the recognition of NANPs by human blood cells is determined by multiple physicochemical parameters such as 3D structure, composition, size, connectivity, and lengths of the single-stranded moieties present in their structures. It further supported the original conclusion of Afonin and his research team that NANPs could serve as a molecular language to allow the researchers to communicate desirable immunological responses to the immune cells. The recent study concluded that the immunological effects of NANPS that are desirable for vaccines and immunotherapies can be enhanced by using RNA as a building block and compacting it into complex structures with 3D shape. Alternatively, when immunological stimulation is unwanted, it can be reduced by either using DNA as a building block or by constructing NANPs that have planar and fibrous shapes. Additional fine-tuning can be achieved by adjusting the connectivity and the overall lengths of the single-stranded regions. 

Interestingly, in the case of NANPs created using 3WJ RNA technology, the structure-activity relationship observed at both the scaffold level and in structures containing scaffold particles functionalized with CpG DNA oligonucleotides [103]. However, structure activity relationship effects were more pronounced after the attachment of the CpG oligonucleotides to their respective scaffolds. In contrast to these findings, the structure-activity relationship of fibrous scaffold NANPs produced using different technology was lost with addition of functional moieties made of siRNA; all siRNA-functionalized fibers had similar pro-inflammatory properties [85]. The differences in the models, study-end-points, type of functional moieties (CpG DNA oligonucleotides [103] vs. siRNA [85]), sequence, materials (DNA vs. RNA), and technology used to prepare these 3WJ and fibrous NANPs could have contributed to the observed differences in their pro-inflammatory response [85,103].

Importantly, in contrast to the traditional nucleic acids such as oligonucleotides, which trigger inflammatory responses at the moment of their exposure, “naked” NANPs are invisible to the immune cells and induce inflammatory cytokines only after the complexation with and delivery into the cells by a vehicle [87]. Another unexpected finding is that TLR7, a protein traditionally cited as the receptor for single-stranded RNA, was shown to control interferon response to both RNA and DNA cubes [87,104]. The open question at the moment is whether and how complexation with or formulation of NANPs using different carriers influences their recognition by TLRs as well as the spectrum and the magnitude of the immunological responses to these particles. Since the immune receptors recognizing nucleic acids are located in various compartments within a cell [35], it is plausible to expect that delivery to a particular intracellular compartment achieved through specific nanocarriers would help to elicit different immune responses to NANPs delivered by these carriers. 

### 3.5. Properties Beneficial for Vaccines and Immunotherapy

NANPs have several properties that make them attractive for vaccines and immunotherapies. They activate antigen-presenting cells to produce type I interferons. This property suggests that NANPs can be used as vaccine adjuvants because interferons support dendritic cell maturation and function [105,106,107]. Since inflammatory responses to adjuvant may vary dramatically between individuals [13], by varying physicochemical properties of NANPs one may personalize a vaccine so that the level and the type of the interferon response is optimal for the given patient. This personalization would allow providing efficacy and avoid immune-mediated adverse effects. A thorough investigation to link specific genomic, transcriptomic, metabolomic and microbiota profiles of a donor to the type and the magnitude of the interferon response would enable personalized selection of optimal NANP-based adjuvants. Type I interferons are proven to have therapeutic effects against viral infections and cancer; they are also considered for the therapy of multiple sclerosis [108,109,110]. Recombinant interferon-alpha, for example, is used in the clinic to treat hepatitis C infections [111] and is the first line of treatment for chronic myeloid leukemia [112]. Despite unarguable benefit of this drug, its systemic distribution is often accompanied by immune-mediated adverse effects such as fever and chills [113]. In addition, some patients develop an antibody to recombinant proteins [114]. Such anti-drug antibodies have a variety of consequences including but not limited to neutralization of the drug product. In the presence of the neutralizing antibodies the drug product loses its efficacy [115]. Protein engineering and modification with polyethylene glycol (PEG) are common strategies to mitigate the risk of immunogenicity of recombinant protein therapeutics [115]. Unlike the original expectations, PEGylation is proven not to be a panacea from the immunogenicity of recombinant protein therapies. There is an increasing number of reports about the immunogenicity of the PEG molecules and rising prevalence of anti-PEG antibodies in the blood of healthy individuals [116,117,118]. These recent concerns dictate the need for alternative solutions to overcome the problems associated with the immunogenicity of recombinant interferon therapeutics. NANPs could potentially address these problems by bypassing the use of recombinant interferons. Using different nanocarriers, NANPs can be delivered to liver or myeloid cells in the blood, and induce hosts’ own type I interferons. For example, Hong et al. demonstrated that DNA and RNA NANPs induce all members of the type I interferon family (interferon alpha, interferon beta, interferon omega) [87]. Targeted delivery of these NANPs to the cells or tissue of interest would enable local interferon induction and bypass systemic toxicity, while body’s own type I interferons would not be immunogenic. More studies are needed to verify this attractive hypothesis.

Another interesting property is the ability of NANPs to induce type III interferons. Specifically, Hong et al. found that DNA and RNA cubes, as well as other DNA and RNA NANPs, induce interferon lambda [87]. This interferon is less potent than type I interferons and provides anti-viral protection at the epithelial barrier with minimal damaging inflammation [119]. Therefore, it can be used to control viral infections at the local level. Similar to the ideas discussed above for the type I interferon, I, therefore, hypothesize that local administration of NANPs may induce the body’s natural response against viral infections via the induction of type III interferons and without the complications associated with recombinant protein therapies. Additionally, interferon lambda is thought to have anti-cancer activity, and, therefore, is widely considered for cancer therapy [120]. Interestingly, despite overlapping functions and unlike type I interferons, the expression of receptors to interferon lambda is limited to specific tissues in that it is expressed at high levels in the liver, prostate, and lung [120]. It is also selectively expressed in keratinocytes and melanocyte, but not in other cell types present in the skin [120]. Therefore, therapy for cancerous lesions in these organs would benefit from type III interferon therapy. It would be interesting to understand whether the NANPs used in the studies by Hong et al., [87] could be engineered to exclusively induce type I or type III interferons. Such knowledge would enable additional applications of NANPs in the prevention and treatment of infectious diseases and cancer.

NANP’s scaffolds were proven to deliver therapeutic oligonucleotides [66,72,77,121]. This modality may allow combining the beneficial immune response with additional therapeutic activities. One area where such complex drugs may be beneficial is cancer, a systemic disorder with many immune pathways dysfunction to form so-called cancer immunity cycle [122]. The combined properties of NANPs’ scaffold to activate antigen-presenting cells and functional moieties to up- or downregulate the expression of immune checkpoint proteins and homing cytokines may help restoring the normal immune function in the affected host. By selecting a nanocarrier for the tumor-specific delivery of NANPs or designing a NANP to deliver cytotoxic drugs, one could also contribute to the induction of immunogenic cell death of cancer cells. Therefore, NANPs technology appears versatile and capable of breaking the cancer immunity cycle at multiple points. More research is needed to verify the utility of these promising modalities in the immunotherapy of cancer.

NANPs split functionality can be created when two individually non-functional NANPs are co-delivered into the same cell where they re-associate to create a fully functional NANP [76,77,123]. This modality provides both off and on switches to NANP-mediated biological responses. When individual NANPs get off-target, the lack of their functionality at the individual level would prevent undesirable side effects. Afonin et al. proposed an interesting concept in which an individual functional NANP is administered as a single therapeutic entity, then another NANP neutralizing the effect of the functional NANP is administered to quench the biological response and thereby avoid avert response [76,77,123]. This controlled “off” property is very attractive in immunotherapy, as it may provide control over the activated immune cells to avoid auto-immunity. For example, a typical shortcoming of the current checkpoint blockade inhibitors is that they unleash the tumor-specific T-cells but do not provide control over what these T-cells do after tumor eradication and result in autoimmune response. Therefore, applying NANPs to quench the activated T-cells after tumor eradication could help to avoid such side effects. In another example of the controlled “off/on” activity, non-functional individually delivered NANPs are combined inside the cell to create a functional material capable of activating or inhibiting the function of cellular proteins. For example, fibers releasing NF-κB decoy oligonucleotides were shown to inhibit NF-kB function only after co-delivery to the target cell [82]. Abnormal NF-kB function is well documented in both solid tumors and hematological malignancies [124,125,126,127], and its inhibition by functional NANPs would, therefore, contribute to the anti-cancer efficacy. Likewise, this property may be found beneficial to control undesirably high induction of TNFα by vaccine adjuvants and avoid necrosis at the site of vaccine administration. Some proof of efficacy of NANPs targeting the activity of NF-κB has already been demonstrated [82]. The promising results observed by Ke et al., warrant more studies to further explore the application of NANPs in this therapeutic area.

### 3.6. Translational Considerations

Translation of NANP-based therapeutics to clinical application requires filling many knowledge gaps. Identifying the critical parameters to allow both efficacy and safety of NANPS would benefit patients receiving NANPs-based therapies. Some of these challenges have been discussed before [45]. Stability in biological matrix, potency, pharmacokinetics (PK) and pharmacodynamics (PD) favorable for the intended application, specificity to the target of interest, exaggerated pharmacology and safety profiles are among the general requirement for drug product in the category of therapeutic nucleic acids [128]. NANPs’ molecular weight and structure are different from those of the traditional TNA. While NANPs versatility and strategies for design of functional NANPs have been studied extensively [46,47,48,49,50,51,52,53,55,57,65,66,67,68,70,71,72,73,74,76,77,78,79,80,81,83,86,91,92,95,96,97,98,100,102,103,121,129,130,131,132,133,134,135,136,137,138,139,140,141,142,143], their off-target toxicity, Absorption, Distribution, Metabolism and Excretion (ADME), PK and PD profiles, are largely unknown as are the abilities to induce anti-NANP immune response (immunogenicity) and break the host tolerance to self-nucleic acids (autoimmune properties). 

Unanswered questions also remain about NANPs local concentration at the injection site, distribution to the systemic circulation after administration through alternative routes; rate and routes of clearance; biodistribution and its dependence on the route of administration, size, secondary structure, conformation, sequences and sequence complementarity, chemical modifications of both the scaffold and functional moieties oligonucleotides; protein binding and MPS clearance; metabolism; general toxicity, reproductive, immune and gene toxicities. All of these questions need to be answered for NANPs alone and in the context of a carrier if a carrier is used. Biocompatibility and immunotoxicity of nanoparticle carriers are determined by their physicochemical properties [14,15,16,17]. It is important to understand how properties of carriers change after complexation with NANPs, and whether or not such complexation creates new toxicities not observed when NANPs and carriers are used separately. When both a NANP and a carrier exhibit overlapping toxicity (e.g., cytokine induction or activation of complement), consideration should be given to the intended application. For example, when the NANP-carrier with overlapping toxicity is used for the systemic administration and non-immune indication, such overlap would likely create a safety issue; therefore, an alternative carrier may be needed. In contrast, when a local administration and vaccine indication is considered, then overlapping properties may be beneficial.

Another common translational hurdle of nanotechnology is the potential contamination with endotoxin [144,145,146]. While challenges with the production of pyrogen-free NANPs have been addressed on a small scale [102], scale-up of NANPs technology to volumes and quantities relevant to clinical use may require further optimization. 

Guo’s lab recently reported a scaled-up automated production of 3WJ RNA nanoparticles [137]. More work is this area is needed to assure the high quality and affordability of NANPs for clinical use. There is an increasing concern regarding the high costs of many cutting-edge medicines, e.g., cellular immunotherapies, that make them unaffordable to patients and affect the revenues of companies producing these products [147,148]. In this context, developing procedures that would allow fast and inexpensive production of pyrogen-free NANPs would further benefit the translation of these materials to the clinic. 

Since NANPs are considered for immunotherapy, detection, and quantification of other innate immunity modulating impurities (IIMIs) (e.g., beta-glucans and flagellin) [149] may also be needed. While these impurities are less potent than endotoxin, in the context of the immunotherapeutic application of NANPs, they may both contribute to efficacy and create safety issues. Investigation of the potential contamination of NANPs with IIMIs other than endotoxin and its contribution to NANPs immunostimulatory properties opens another new area of future translational research on these nanomaterials.

## 4. Outlook into the Future

In order to bring NANP technology to the clinic, studies investigating their adsorption, distribution, metabolism, excretion, toxicities (general, gene, immune and reproductive) in two animal species a rodent (e.g., rat) and non-rodent (e.g., non-human primate), and hybridization-dependent toxicities in the context of exaggerated pharmacology are needed along with verification of the proposed efficacy. Scale-up and optimization of manufacturing procedures to produce affordable, pyrogen-free NANPs are also required. Understanding the potential long-term effects including autoimmunity is necessary. The development of bioanalytical assays along with standardized methods for NANPs characterization would further advance this technology toward clinical applications. Since NANPs synthesis is well controlled, applying bioinformatics and artificial intelligence tools may speed up both the design and prioritization of various NANPs structures for preclinical studies as well as assist with identifying promising indications. Mechanistic studies uncovering NANPs interaction with various cell types, mechanisms of NANPs immune recognition, and understanding the potential of this technology for diagnostics field are beneficial. As the production of these materials moves toward large-scale, understanding their biodegradability and environmental effects would also be needed. These are just a few ideas for what NANPs field would be facing in the upcoming decade.

## References

[1] Gao Y.L., Zhai J.H., Chai Y.F. (2018). Recent Advances in the Molecular Mechanisms Underlying Pyroptosis in Sepsis. Mediat. Inflamm..

[2] Dickmann P., Bauer M. (2019). Sepsis 2019-New Trends and Their Implications for Multiple Trauma Patients. Z. Orthop. Unf..

[3] Sattler S. (2017). The Role of the Immune System Beyond the Fight Against Infection. Adv. Exp. Med. Biol..

[4] Zolnik B.S., Gonzalez-Fernandez A., Sadrieh N., Dobrovolskaia M.A. (2010). Nanoparticles and the immune system. Endocrinology.

[5] Coutinho A.E., Chapman K.E. (2011). The anti-inflammatory and immunosuppressive effects of glucocorticoids, recent developments and mechanistic insights. Mol. Cell Endocrinol..

[6] Garcia-Martinez E., Smith M., Buque A., Aranda F., de la Pena F.A., Ivars A., Canovas M.S., Conesa M.A.V., Fucikova J., Spisek R. (2018). Trial Watch: Immunostimulation with recombinant cytokines for cancer therapy. Oncoimmunology.

[7] Horwitz D.A., Fahmy T.M., Piccirillo C.A., La Cava A. (2019). Rebalancing Immune Homeostasis to Treat Autoimmune Diseases. Trends Immunol..

[8] Abbas A.K., Lichtman A.H., Pillai S. (2018). Cellular and Molecular Immunology.

[9] Andrews L.P., Yano H., Vignali D.A.A. (2019). Inhibitory receptors and ligands beyond PD-1, PD-L1 and CTLA-4: Breakthroughs or backups. Nat. Immunol..

[10] Zindel J., Kubes P. (2019). DAMPs, PAMPs, and LAMPs in Immunity and Sterile Inflammation. Annu. Rev. Pathol..

[11] Reichhardt M.P., Meri S. (2018). Intracellular complement activation-An alarm raising mechanism?. Semin. Immunol..

[12] Bastola R., Noh G., Keum T., Bashyal S., Seo J.E., Choi J., Oh Y., Cho Y., Lee S. (2017). Vaccine adjuvants: Smart components to boost the immune system. Arch. Pharm. Res..

[13] Duffy D. (2018). Milieu interieur: Defining the boundaries of a healthy immune response for improved vaccination strategies. Hum. Vaccin. Immunother..

[14] Dobrovolskaia M.A., McNeil S.E. (2007). Immunological properties of engineered nanomaterials. Nat. Nanotechnol..

[15] Dobrovolskaia M.A., Shurin M., Shvedova A.A. (2016). Current understanding of interactions between nanoparticles and the immune system. Toxicol. Appl. Pharm..

[16] Boraschi D., Costantino L., Italiani P. (2012). Interaction of nanoparticles with immunocompetent cells: Nanosafety considerations. Nanomedicine.

[17] Boraschi D., Italiani P., Palomba R., Decuzzi P., Duschl A., Fadeel B., Moghimi S.M. (2017). Nanoparticles and innate immunity: New perspectives on host defence. Semin. Immunol..

[18] Banday A.H., Jeelani S., Hruby V.J. (2015). Cancer vaccine adjuvants–recent clinical progress and future perspectives. Immunopharmacol. Immunotoxicol..

[19] Geynisman D.M., Chien C.R., Smieliauskas F., Shen C., Shih Y.C. (2014). Economic evaluation of therapeutic cancer vaccines and immunotherapy: A systematic review. Hum. Vaccin. Immunother..

[20] Song Q., Zhang C.D., Wu X.H. (2018). Therapeutic cancer vaccines: From initial findings to prospects. Immunol. Lett..

[21] Cohen K.W., Frahm N. (2017). Current views on the potential for development of a HIV vaccine. Expert. Opin. Biol..

[22] Lundstrom K. (2015). RNA-based drugs and vaccines. Expert Rev. Vaccines.

[23] Pardi N., Hogan M.J., Porter F.W., Weissman D. (2018). mRNA vaccines-a new era in vaccinology. Nat. Rev. Drug Discov..

[24] Kelly H.G., Kent S.J., Wheatley A.K. (2019). Immunological basis for enhanced immunity of nanoparticle vaccines. Expert Rev. Vaccines.

[25] Pati R., Shevtsov M., Sonawane A. (2018). Nanoparticle Vaccines Against Infectious Diseases. Front. Immunol..

[26] Sulczewski F.B., Liszbinski R.B., Romao P.R.T., Rodrigues Junior L.C. (2018). Nanoparticle vaccines against viral infections. Arch. Virol..

[27] Zhao L., Seth A., Wibowo N., Zhao C.X., Mitter N., Yu C., Middelberg A.P. (2014). Nanoparticle vaccines. Vaccine.

[28] Grimaldi A.M., Incoronato M., Salvatore M., Soricelli A. (2017). Nanoparticle-based strategies for cancer immunotherapy and immunodiagnostics. Nanomedicine.

[29] Hong E., Dobrovolskaia M.A. (2019). Addressing barriers to effective cancer immunotherapy with nanotechnology: Achievements, challenges, and roadmap to the next generation of nanoimmunotherapeutics. Adv. Drug Deliv. Rev..

[30] Milling L., Zhang Y., Irvine D.J. (2017). Delivering safer immunotherapies for cancer. Adv. Drug Deliv. Rev..

[31] Oberli M.A., Reichmuth A.M., Dorkin J.R., Mitchell M.J., Fenton O.S., Jaklenec A., Anderson D.G., Langer R., Blankschtein D. (2017). Lipid Nanoparticle Assisted mRNA Delivery for Potent Cancer Immunotherapy. Nano Lett..

[32] Shao K., Singha S., Clemente-Casares X., Tsai S., Yang Y., Santamaria P. (2015). Nanoparticle-based immunotherapy for cancer. ACS Nano.

[33] Yoon H.Y., Selvan S.T., Yang Y., Kim M.J., Yi D.K., Kwon I.C., Kim K. (2018). Engineering nanoparticle strategies for effective cancer immunotherapy. Biomaterials.

[34] Jordheim L.P., Durantel D., Zoulim F., Dumontet C. (2013). Advances in the development of nucleoside and nucleotide analogues for cancer and viral diseases. Nat. Rev. Drug Discov..

[35] Dobrovolskaia M.A., McNeil S.E. (2015). Immunological and hematological toxicities challenging clinical translation of nucleic acid-based therapeutics. Expert Opin. Biol..

[36] Watts J.K., Brown R.H., Khvorova A. (2019). Nucleic Acid Therapeutics for Neurological Diseases. Neurotherapeutics.

[37] Yadav S., Shekhawat M., Jahagirdar D., Kumar Sharma N. (2017). Natural and artificial small RNAs: A promising avenue of nucleic acid therapeutics for cancer. Cancer Biol. Med..

[38] Henry S.P., Templin M.V., Gillett N., Rojko J., Levin A.A. (1999). Correlation of toxicity and pharmacokinetic properties of a phosphorothioate oligonucleotide designed to inhibit ICAM-1. Toxicol. Pathol..

[39] Keefe A.D., Pai S., Ellington A. (2010). Aptamers as therapeutics. Nat. Rev. Drug Discov..

[40] Levin A.A. (1999). A review of the issues in the pharmacokinetics and toxicology of phosphorothioate antisense oligonucleotides. Biochim. Biophys. Acta.

[41] Monteith D.K., Horner M.J., Gillett N.A., Butler M., Geary R., Burckin T., Ushiro-Watanabe T., Levin A.A. (1999). Evaluation of the renal effects of an antisense phosphorothioate oligodeoxynucleotide in monkeys. Toxicol. Pathol..

[42] Monteith D.K., Levin A.A. (1999). Synthetic oligonucleotides: The development of antisense therapeutics. Toxicol. Pathol..

[43] Lindow M., Vornlocher H.P., Riley D., Kornbrust D.J., Burchard J., Whiteley L.O., Kamens J., Thompson J.D., Nochur S., Younis H. (2012). Assessing unintended hybridization-induced biological effects of oligonucleotides. Nat. Biotechnol..

[44] Alnylam Alnylam Announces Approval of GIVLAARI™ (givosiran) by the US Food and Drug Administration (FDA). https://www.businesswire.com/news/home/20191120005849/en/Alnylam-Announces-Approval-GIVLAARI%E2%84%A2-givosiran-U.S.-Food.

[45] Dobrovolskaia M.A. (2016). Self-assembled DNA/RNA nanoparticles as a new generation of therapeutic nucleic acids: Immunological compatibility and other translational considerations. DNA RNA Nanotechnol..

[46] Afonin K.A., Leontis N.B. (2006). Generating new specific RNA interaction interfaces using C-loops. J. Am. Chem. Soc..

[47] Afonin K.A., Cieply D.J., Leontis N.B. (2008). Specific RNA self-assembly with minimal paranemic motifs. J. Am. Chem. Soc..

[48] Grabow W.W., Zakrevsky P., Afonin K.A., Chworos A., Shapiro B.A., Jaeger L. (2011). Self-assembling RNA nanorings based on RNAI/II inverse kissing complexes. Nano Lett..

[49] Afonin K.A., Kireeva M., Grabow W.W., Kashlev M., Jaeger L., Shapiro B.A. (2012). Co-transcriptional assembly of chemically modified RNA nanoparticles functionalized with siRNAs. Nano Lett..

[50] Afonin K.A., Desai R., Viard M., Kireeva M.L., Bindewald E., Case C.L., Maciag A.E., Kasprzak W.K., Kim T., Sappe A. (2014). Co-transcriptional production of RNA-DNA hybrids for simultaneous release of multiple split functionalities. Nucleic Acids Res..

[51] Afonin K.A., Kasprzak W., Bindewald E., Puppala P.S., Diehl A.R., Hall K.T., Kim T.J., Zimmermann M.T., Jernigan R.L., Jaeger L. (2014). Computational and experimental characterization of RNA cubic nanoscaffolds. Methods.

[52] Afonin K.A., Kasprzak W.K., Bindewald E., Kireeva M., Viard M., Kashlev M., Shapiro B.A. (2014). In silico design and enzymatic synthesis of functional RNA nanoparticles. Acc. Chem. Res..

[53] Afonin K.A., Viard M., Koyfman A.Y., Martins A.N., Kasprzak W.K., Panigaj M., Desai R., Santhanam A., Grabow W.W., Jaeger L. (2014). Multifunctional RNA nanoparticles. Nano Lett..

[54] Parlea L., Bindewald E., Sharan R., Bartlett N., Moriarty D., Oliver J., Afonin K.A., Shapiro B.A. (2016). Ring Catalog: A resource for designing self-assembling RNA nanostructures. Methods.

[55] Stewart J.M., Viard M., Subramanian H.K., Roark B.K., Afonin K.A., Franco E. (2016). Programmable RNA microstructures for coordinated delivery of siRNAs. Nanoscale.

[56] Bui M.N., Brittany Johnson M., Viard M., Satterwhite E., Martins A.N., Li Z., Marriott I., Afonin K.A., Khisamutdinov E.F. (2017). Versatile RNA tetra-U helix linking motif as a toolkit for nucleic acid nanotechnology. Nanomedicine.

[57] Jasinski D., Haque F., Binzel D.W., Guo P. (2017). Advancement of the Emerging Field of RNA Nanotechnology. ACS Nano.

[58] Guo P. (2010). The emerging field of RNA nanotechnology. Nat. Nanotechnol..

[59] Rothemund P.W.K. (2006). Folding DNA to create nanoscale shapes and patterns. Nature.

[60] Rothemund P.W.K., Andersen E.S. (2012). Nanotechnology: The importance of being modular. Nature.

[61] Mei Q., Wei X., Su F., Liu Y., Youngbull C., Johnson R., Lindsay S., Yan H., Meldrum D. (2011). Stability of DNA origami nanoarrays in cell lysate. Nano Lett..

[62] Schuller V.J., Heidegger S., Sandholzer N., Nickels P.C., Suhartha N.A., Endres S., Bourquin C., Liedl T. (2011). Cellular immunostimulation by CpG-sequence-coated DNA origami structures. ACS Nano.

[63] Mikkilä J., Eskelinen A.-P., Niemelä E.H., Linko V., Frilander M.J., Törmä P., Kostiainen M.A. (2014). Virus-encapsulated DNA origami nanostructures for cellular delivery. Nano Lett..

[64] Zhang Q., Jiang Q., Li N., Dai L., Liu Q., Song L., Wang J., Li Y., Tian J., Ding B. (2014). DNA origami as an in vivo drug delivery vehicle for cancer therapy. ACS Nano.

[65] Shu D., Khisamutdinov E.F., Zhang L., Guo P. (2014). Programmable folding of fusion RNA in vivo and in vitro driven by pRNA 3WJ motif of phi29 DNA packaging motor. Nucleic Acids Res..

[66] Shu Y., Haque F., Shu D., Li W., Zhu Z., Kotb M., Lyubchenko Y., Guo P. (2013). Fabrication of 14 different RNA nanoparticles for specific tumor targeting without accumulation in normal organs. RNA.

[67] Shu Y., Pi F., Sharma A., Rajabi M., Haque F., Shu D., Leggas M., Evers B.M., Guo P. (2014). Stable RNA nanoparticles as potential new generation drugs for cancer therapy. Adv. Drug Deliv. Rev..

[68] Shu Y., Shu D., Haque F., Guo P. (2013). Fabrication of pRNA nanoparticles to deliver therapeutic RNAs and bioactive compounds into tumor cells. Nat. Protoc..

[69] Binzel D.W., Khisamutdinov E., Vieweger M., Ortega J., Li J., Guo P. (2016). Mechanism of three-component collision to produce ultrastable pRNA three-way junction of Phi29 DNA-packaging motor by kinetic assessment. RNA.

[70] Grabow W.W., Jaeger L. (2014). RNA self-assembly and RNA nanotechnology. Acc. Chem. Res..

[71] Afonin K.A., Lin Y.P., Calkins E.R., Jaeger L. (2012). Attenuation of loop-receptor interactions with pseudoknot formation. Nucleic Acids Res..

[72] Afonin K.A., Viard M., Kagiampakis I., Case C.L., Dobrovolskaia M.A., Hofmann J., Vrzak A., Kireeva M., Kasprzak W.K., KewalRamani V.N. (2015). Triggering of RNA interference with RNA-RNA, RNA-DNA, and DNA-RNA nanoparticles. ACS Nano.

[73] Afonin K.A., Viard M., Martins A.N., Lockett S.J., Maciag A.E., Freed E.O., Heldman E., Jaeger L., Blumenthal R., Shapiro B.A. (2013). Activation of different split functionalities on re-association of RNA-DNA hybrids. Nat. Nanotechnol..

[74] Afonin K.A., Viard M., Tedbury P., Bindewald E., Parlea L., Howington M., Valdman M., Johns-Boehme A., Brainerd C., Freed E.O. (2016). The Use of Minimal RNA Toeholds to Trigger the Activation of Multiple Functionalities. Nano Lett..

[75] Bindewald E., Afonin K.A., Viard M., Zakrevsky P., Kim T., Shapiro B.A. (2016). Multistrand Structure Prediction of Nucleic Acid Assemblies and Design of RNA Switches. Nano Lett..

[76] Chandler M., Afonin K.A. (2019). Smart-Responsive Nucleic Acid Nanoparticles (NANPs) with the Potential to Modulate Immune Behavior. Nanomaterials.

[77] Chandler M., Lyalina T., Halman J., Rackley L., Lee L., Dang D., Ke W., Sajja S., Woods S., Acharya S. (2018). Broccoli Fluorets: Split Aptamers as a User-Friendly Fluorescent Toolkit for Dynamic RNA Nanotechnology. Molecules.

[78] Feng L., Li S.K., Liu H., Liu C.Y., LaSance K., Haque F., Shu D., Guo P. (2014). Ocular delivery of pRNA nanoparticles: Distribution and clearance after subconjunctival injection. Pharm. Res..

[79] Halman J.R., Kim K.T., Gwak S.J., Pace R., Johnson M.B., Chandler M.R., Rackley L., Viard M., Marriott I., Lee J.S. (2019). A cationic amphiphilic co-polymer as a carrier of nucleic acid nanoparticles (Nanps) for controlled gene silencing, immunostimulation, and biodistribution. Nanomedicine.

[80] Halman J.R., Afonin K.A. (2019). Editorial for the Special Issue on “Nucleic Acid Architectures for Therapeutics, Diagnostics, Devices and Materials”. Nanomaterials.

[81] Halman J.R., Satterwhite E., Roark B., Chandler M., Viard M., Ivanina A., Bindewald E., Kasprzak W.K., Panigaj M., Bui M.N. (2017). Functionally-interdependent shape-switching nanoparticles with controllable properties. Nucleic Acids Res..

[82] Ke W., Hong E., Saito R.F., Rangel M.C., Wang J., Viard M., Richardson M., Khisamutdinov E.F., Panigaj M., Dokholyan N.V. (2019). RNA-DNA fibers and polygons with controlled immunorecognition activate RNAi, FRET and transcriptional regulation of NF-kappaB in human cells. Nucleic Acids Res..

[83] Kireeva M.L., Afonin K.A., Shapiro B.A., Kashlev M. (2017). Cotranscriptional Production of Chemically Modified RNA Nanoparticles. Methods Mol. Biol..

[84] Parlea L., Puri A., Kasprzak W., Bindewald E., Zakrevsky P., Satterwhite E., Joseph K., Afonin K.A., Shapiro B.A. (2016). Cellular Delivery of RNA Nanoparticles. ACS Comb. Sci..

[85] Rackley L., Stewart J.M., Salotti J., Krokhotin A., Shah A., Halman J.R., Juneja R., Smollett J., Lee L., Roark K. (2018). RNA Fibers as Optimized Nanoscaffolds for siRNA Coordination and Reduced Immunological Recognition. Adv. Funct. Mater..

[86] Haque F., Pi F., Zhao Z., Gu S., Hu H., Yu H., Guo P. (2018). RNA versatility, flexibility, and thermostability for practice in RNA nanotechnology and biomedical applications. Wiley Interdiscip. Rev. RNA.

[87] Hong E., Halman J.R., Shah A.B., Khisamutdinov E.F., Dobrovolskaia M.A., Afonin K.A. (2018). Structure and Composition Define Immunorecognition of Nucleic Acid Nanoparticles. Nano Lett..

[88] Kim T., Afonin K.A., Viard M., Koyfman A.Y., Sparks S., Heldman E., Grinberg S., Linder C., Blumenthal R.P., Shapiro B.A. (2013). In Silico, In Vitro, and In Vivo Studies Indicate the Potential Use of Bolaamphiphiles for Therapeutic siRNAs Delivery. Mol. Nucleic Acids.

[89] Sharma V.K., Watts J.K. (2015). Oligonucleotide therapeutics: Chemistry, delivery and clinical progress. Future Med. Chem..

[90] Lee T., Yagati A.K., Pi F., Sharma A., Choi J.W., Guo P. (2015). Construction of RNA-Quantum Dot Chimera for Nanoscale Resistive Biomemory Application. ACS Nano.

[91] Afonin K.A., Bindewald E., Kireeva M., Shapiro B.A. (2015). Computational and experimental studies of reassociating RNA/DNA hybrids containing split functionalities. Methods Enzym..

[92] Afonin K.A., Schultz D., Jaeger L., Gwinn E., Shapiro B.A. (2015). Silver nanoclusters for RNA nanotechnology: Steps towards visualization and tracking of RNA nanoparticle assemblies. Methods Mol. Biol..

[93] Cruz-Acuna M., Halman J.R., Afonin K.A., Dobson J., Rinaldi C. (2018). Magnetic nanoparticles loaded with functional RNA nanoparticles. Nanoscale.

[94] Juneja R., Lyles Z., Vadarevu H., Afonin K.A., Vivero-Escoto J.L. (2019). Multimodal Polysilsesquioxane Nanoparticles for Combinatorial Therapy and Gene Delivery in Triple-Negative Breast Cancer. Acs Appl. Mater. Interfaces.

[95] Haque F., Shu D., Shu Y., Shlyakhtenko L.S., Rychahou P.G., Evers B.M., Guo P. (2012). Ultrastable synergistic tetravalent RNA nanoparticles for targeting to cancers. Nano Today.

[96] Shu D., Shu Y., Haque F., Abdelmawla S., Guo P. (2011). Thermodynamically stable RNA three-way junction for constructing multifunctional nanoparticles for delivery of therapeutics. Nat. Nanotechnol..

[97] Abdelmawla S., Guo S., Zhang L., Pulukuri S.M., Patankar P., Conley P., Trebley J., Guo P., Li Q.X. (2011). Pharmacological characterization of chemically synthesized monomeric phi29 pRNA nanoparticles for systemic delivery. Mol. Ther..

[98] Lee H., Lytton-Jean A.K., Chen Y., Love K.T., Park A.I., Karagiannis E.D., Sehgal A., Querbes W., Zurenko C.S., Jayaraman M. (2012). Molecularly self-assembled nucleic acid nanoparticles for targeted in vivo siRNA delivery. Nat. Nanotechnol..

[99] Glover J.M., Leeds J.M., Mant T.G., Amin D., Kisner D.L., Zuckerman J.E., Geary R.S., Levin A.A., Shanahan W.R. (1997). Phase I safety and pharmacokinetic profile of an intercellular adhesion molecule-1 antisense oligodeoxynucleotide (ISIS 2302). J. Pharm. Exp..

[100] Zhou J., Shu Y., Guo P., Smith D.D., Rossi J.J. (2011). Dual functional RNA nanoparticles containing phi29 motor pRNA and anti-gp120 aptamer for cell-type specific delivery and HIV-1 inhibition. Methods.

[101] PrabhuDas M.R., Baldwin C.L., Bollyky P.L., Bowdish D.M.E., Drickamer K., Febbraio M., Herz J., Kobzik L., Krieger M., Loike J. (2017). A Consensus Definitive Classification of Scavenger Receptors and Their Roles in Health and Disease. J. Immunol..

[102] Afonin K.A., Grabow W.W., Walker F.M., Bindewald E., Dobrovolskaia M.A., Shapiro B.A., Jaeger L. (2011). Design and self-assembly of siRNA-functionalized RNA nanoparticles for use in automated nanomedicine. Nat. Protoc..

[103] Khisamutdinov E.F., Li H., Jasinski D.L., Chen J., Fu J., Guo P. (2014). Enhancing immunomodulation on innate immunity by shape transition among RNA triangle, square and pentagon nanovehicles. Nucleic Acids Res..

[104] Hong E., Halman J.R., Shah A., Cedrone E., Truong N., Afonin K.A., Dobrovolskaia M.A. (2019). Toll-Like Receptor-Mediated Recognition of Nucleic Acid Nanoparticles (NANPs) in Human Primary Blood Cells. Molecules.

[105] Radvanyi L.G., Banerjee A., Weir M., Messner H. (1999). Low levels of interferon-alpha induce CD86 (B7.2) expression and accelerates dendritic cell maturation from human peripheral blood mononuclear cells. Scand. J. Immunol..

[106] Tam M.A., Wick M.J. (2009). MyD88 and interferon-alpha/beta are differentially required for dendritic cell maturation but dispensable for development of protective memory against Listeria. Immunology.

[107] Trepiakas R., Pedersen A.E., Met O., Svane I.M. (2009). Addition of interferon-alpha to a standard maturation cocktail induces CD38 up-regulation and increases dendritic cell function. Vaccine.

[108] Floros T., Tarhini A.A. (2015). Anticancer Cytokines: Biology and Clinical Effects of Interferon-alpha2, Interleukin (IL)-2, IL-15, IL-21, and IL-12. Semin. Oncol..

[109] Cheknev S.B., Kobyakina N.A., Mezentseva M.V., Skvortsova V.I. (2001). Long-Term Study of Interferon System State in Patients with Multiple Sclerosis Received the Individual Immune Therapy with Human Recombinant IFN-alpha. Russ. J. Immunol..

[110] Bongioanni M.R., Durelli L., Ferrero B., Imperiale D., Oggero A., Verdun E., Aimo G., Pagni R., Geuna M., Bergamasco B. (1996). Systemic high-dose recombinant-alpha-2a-interferon therapy modulates lymphokine production in multiple sclerosis. J. Neurol. Sci..

[111] Rong L., Perelson A.S. (2009). Modeling HIV persistence, the latent reservoir, and viral blips. J. Biol..

[112] Kujawski L.A., Talpaz M. (2007). The role of interferon-alpha in the treatment of chronic myeloid leukemia. Cytokine Growth Factor Rev..

[113] Filipi M.L., Beavin J., Brillante R.T., Costello K., Hartley G.C., Hartley K., Namey M., O’Leary S., Remington G. (2014). Nurses’ perspective on approaches to limit flu-like symptoms during interferon therapy for multiple sclerosis. Int. J. Ms Care.

[114] Rosenberg A.S. (2003). Immunogenicity of biological therapeutics: A hierarchy of concerns. Dev. Biol. (Basel).

[115] Baker M.P., Reynolds H.M., Lumicisi B., Bryson C.J. (2010). Immunogenicity of protein therapeutics: The key causes, consequences and challenges. Self/Nonself.

[116] Chang C.J., Chen C.H., Chen B.M., Su Y.C., Chen Y.T., Hershfield M.S., Lee M.M., Cheng T.L., Chen Y.T., Roffler S.R. (2017). A genome-wide association study identifies a novel susceptibility locus for the immunogenicity of polyethylene glycol. Nat Commun.

[117] Chen B.M., Su Y.C., Chang C.J., Burnouf P.A., Chuang K.H., Chen C.H., Cheng T.L., Chen Y.T., Wu J.Y., Roffler S.R. (2016). Measurement of Pre-Existing IgG and IgM Antibodies against Polyethylene Glycol in Healthy Individuals. Anal. Chem..

[118] Hsieh Y.C., Wang H.E., Lin W.W., Roffler S.R., Cheng T.C., Su Y.C., Li J.J., Chen C.C., Huang C.H., Chen B.M. (2018). Pre-existing anti-polyethylene glycol antibody reduces the therapeutic efficacy and pharmacokinetics of PEGylated liposomes. Theranostics.

[119] Lazear H.M., Schoggins J.W., Diamond M.S. (2019). Shared and Distinct Functions of Type I and Type III Interferons. Immunity.

[120] Stiff A., Carson W. (2015). Investigations of interferon-lambda for the treatment of cancer. J. Innate Immun..

[121] Shu D., Li H., Shu Y., Xiong G., Carson W.E., Haque F., Xu R., Guo P. (2015). Systemic Delivery of Anti-miRNA for Suppression of Triple Negative Breast Cancer Utilizing RNA Nanotechnology. ACS Nano.

[122] Chen D.S., Mellman I. (2013). Oncology meets immunology: The cancer-immunity cycle. Immunity.

[123] Zakrevsky P., Parlea L., Viard M., Bindewald E., Afonin K.A., Shapiro B.A., Martins A.N., Ke W., Jawahar V., Striplin M. (2017). Preparation of a Conditional RNA Switch Intracellular Reassociation of RNA-DNA Hybrids that Activates RNAi in HIV-Infected Cells Cotranscriptional Production of Chemically Modified RNA Nanoparticles Correction: Programmable RNA microstructures for coordinated delivery of siRNAs Functionally-interdependent shape-switching nanoparticles with controllable properties. Methods Mol. Biol..

[124] Dolcet X., Llobet D., Pallares J., Matias-Guiu X. (2005). NF-kB in development and progression of human cancer. Virchows Arch..

[125] Li F., Zhang J., Arfuso F., Chinnathambi A., Zayed M.E., Alharbi S.A., Kumar A.P., Ahn K.S., Sethi G. (2015). NF-kappaB in cancer therapy. Arch. Toxicol..

[126] Pramanik K.C., Makena M.R., Bhowmick K., Pandey M.K. (2018). Advancement of NF-kappaB Signaling Pathway: A Novel Target in Pancreatic Cancer. Int. J. Mol. Sci..

[127] Tilborghs S., Corthouts J., Verhoeven Y., Arias D., Rolfo C., Trinh X.B., van Dam P.A. (2017). The role of Nuclear Factor-kappa B signaling in human cervical cancer. Crit. Rev. Oncol. Hematol..

[128] Kornbrust D., Cavagnaro J., Levin A., Foy J., Pavco P., Gamba-Vitalo C., Guimond A. (2013). Oligo safety working group exaggerated pharmacology subcommittee consensus document. Nucleic Acids.

[129] Afonin K.A., Bindewald E., Yaghoubian A.J., Voss N., Jacovetty E., Shapiro B.A., Jaeger L. (2010). In vitro assembly of cubic RNA-based scaffolds designed in silico. Nat. Nanotechnol..

[130] Afonin K.A., Danilov E.O., Novikova I.V., Leontis N.B. (2008). TokenRNA: A new type of sequence-specific, label-free fluorescent biosensor for folded RNA molecules. Chembiochem.

[131] Guo P., Shu Y., Binzel D., Cinier M. (2012). Synthesis, conjugation, and labeling of multifunctional pRNA nanoparticles for specific delivery of siRNA, drugs, and other therapeutics to target cells. Methods Mol. Biol..

[132] Guo S., Li H., Ma M., Fu J., Dong Y., Guo P. (2017). Size, Shape, and Sequence-Dependent Immunogenicity of RNA Nanoparticles. Mol. Ther..

[133] Guo S., Piao X., Li H., Guo P. (2018). Methods for construction and characterization of simple or special multifunctional RNA nanoparticles based on the 3WJ of phi29 DNA packaging motor. Methods.

[134] Gupta K., Afonin K.A., Viard M., Herrero V., Kasprzak W., Kagiampakis I., Kim T., Koyfman A.Y., Puri A., Stepler M. (2015). Bolaamphiphiles as carriers for siRNA delivery: From chemical syntheses to practical applications. J. Control. Release.

[135] Haque F., Xu C., Jasinski D.L., Li H., Guo P. (2017). Using Planar Phi29 pRNA Three-Way Junction to Control Size and Shape of RNA Nanoparticles for Biodistribution Profiling in Mice. Methods Mol. Biol..

[136] Haque F., Zhang H., Wang S., Chang C.L., Savran C., Guo P. (2018). Methods for Single-Molecule Sensing and Detection Using Bacteriophage Phi29 DNA Packaging Motor. Methods Mol. Biol..

[137] Jasinski D.L., Binzel D.W., Guo P. (2019). One-Pot Production of RNA Nanoparticles via Automated Processing and Self-Assembly. ACS Nano.

[138] Jasinski D.L., Khisamutdinov E.F., Lyubchenko Y.L., Guo P. (2014). Physicochemically tunable polyfunctionalized RNA square architecture with fluorogenic and ribozymatic properties. ACS Nano.

[139] Jasinski D.L., Li H., Guo P. (2018). The Effect of Size and Shape of RNA Nanoparticles on Biodistribution. Mol. Ther..

[140] Jasinski D.L., Yin H., Li Z., Guo P. (2018). Hydrophobic Effect from Conjugated Chemicals or Drugs on In Vivo Biodistribution of RNA Nanoparticles. Hum. Gene.

[141] Khisamutdinov E.F., Bui M.N., Jasinski D., Zhao Z., Cui Z., Guo P. (2015). Simple Method for Constructing RNA Triangle, Square, Pentagon by Tuning Interior RNA 3WJ Angle from 60 degrees to 90 degrees or 108 degrees. Methods Mol. Biol..

[142] Krishnan Y., Bathe M., Wamhoff E.C., Banal J.L., Bricker W.P., Shepherd T.R., Parsons M.F., Veneziano R., Stone M.B., Jun H. (2012). Designer nucleic acids to probe and program the cell.Programming Structured DNA Assemblies to Probe Biophysical Processes Bioapplications of DNA nanotechnology at the solid-liquid interface. Trends Cell Biol..

[143] Zhang H., Pi F., Shu D., Vieweger M., Guo P. (2015). Using RNA nanoparticles with thermostable motifs and fluorogenic modules for real-time detection of RNA folding and turnover in prokaryotic and eukaryotic cells. Methods Mol. Biol..

[144] Dobrovolskaia M.A., Germolec D.R., Weaver J.L. (2009). Evaluation of nanoparticle immunotoxicity. Nat. Nanotechnol..

[145] Dobrovolskaia M.A., McNeil S.E. (2013). Understanding the correlation between in vitro and in vivo immunotoxicity tests for nanomedicines. J. Control. Release.

[146] Dobrovolskaia M.A. (2015). Pre-clinical immunotoxicity studies of nanotechnology-formulated drugs: Challenges, considerations and strategy. J. Control. Release.

[147] Smith A.G. The Cost of Drugs for Rare Diseases Is Threatening the U.S. Health Care System. https://hbr.org/2017/04/the-cost-of-drugs-for-rare-diseases-is-threatening-the-u-s-health-care-system.

[148] Jena A.B., Kee R., Baumgardner J.R., Ma Q., Zhang J. Making Life-Saving Medical Treatments More Affordable. https://hbr.org/2019/10/making-life-saving-medical-treatments-more-affordable.

[149] Haile L.A., Puig M., Kelley-Baker L., Verthelyi D. (2015). Detection of innate immune response modulating impurities in therapeutic proteins. PLoS ONE.

